# Identification of Potential Exosomal miRNA-mRNA Regulatory Network Relevant to Tuberculous-Associated Lung Cancer

**DOI:** 10.3390/biomedicines14092060

**Published:** 2026-09-14

**Authors:** Hyun-Jung Kang, Goeun Park, Yoonki Hong, Byung-Chul Kim, Hyowon Park, Jae Jun Lee, Seung-Ho Shin, Ji Young Hong

**Affiliations:** 1 Institute of New Frontier Research Team, Hallym University College of Medicine, Chuncheon 24253, Republic of Korea; kangsang8106@naver.com (H.-J.K.); rhdms0608@gmail.com (G.P.); 2 Department of Biochemistry, College of Natural Sciences, Kangwon National University, Chuncheon 24341, Republic of Korea; bckim@kangwon.ac.kr; 3 Department of Internal Medicine, Kangwon National University Hospital, School of Medicine, Kangwon National University, Chuncheon 24289, Republic of Korea; h-doctor@hanmail.net; 4 Integrative Molecular and Biomedical Sciences, College of Biomedical Science, Institute of Bioscience & Biotechnology, Kangwon National University, Chuncheon 24341, Republic of Korea; hyowon@kangwon.ac.kr; 5 Department of Biomedical Informatics, Chuncheon Sacred Heart Hospital, Chuncheon 24253, Republic of Korea; 6 Division of Pulmonary, Allergy and Critical Care Medicine, Department of Internal Medicine, Chuncheon Sacred Heart Hospital, Hallym University Medical Center, Chuncheon 24253, Republic of Korea

**Keywords:** tuberculosis, lung cancer, miRNA-mRNA regulation

## Abstract

**Background/Objectives**: Tuberculosis is a risk factor for lung cancer. Previous studies have demonstrated that miRNAs derived from tuberculous pleural effusion (TPE) exosomes are involved in lung cancer progression. This study aimed to identify potential miRNA–mRNA regulatory networks associated with TPE-derived exosome exposure in the context of lung cancer. **Methods**: Exploratory differential expression analysis was performed using RNA-seq data from TPE-derived exosomes to select candidate miRNAs showing differential expression patterns. In an in vivo mouse xenograft tumor model, TPE-derived exosomes were administered intratumorally, followed by RNA-seq analysis to select candidate genes showing differential expression patterns. Predicted target genes of the candidate miRNAs were then overlapped with the candidate genes from the xenograft analysis to obtain overlapping common genes (OCGs). The regulation of these genes by candidate miRNAs was validated through in vitro experiments, and a miRNA-mRNA network was constructed using Cytoscape. **Results**: Exploratory analyses suggested differential expression patterns in 27 candidate miRNAs (19 showing increased and 8 showing decreased expression) in TPE-derived exosomes and 2340 candidate genes in xenograft tissues. A total of 4715 and 700 target genes were predicted for candidate miRNAs using miRDB and miRTarBase, respectively. Following a stepwise filtering strategy, five candidate target genes (*RRAS2*, *RAC1*, *RAP1B*, *NOTCH2*, and *SMARCA4*) were prioritized from the overlapping common genes (OCGs). The seven candidate miRNAs predicted to regulate these genes were subsequently used to prioritize a candidate 7-miRNA–5-mRNA regulatory network for further investigation. **Conclusions**: This study prioritized a candidate miRNA–mRNA regulatory network associated with TPE-derived exosome exposure in lung cancer models, providing a basis for further investigation in larger clinical cohorts and more physiologically relevant models.

## 1. Introduction

Lung cancer is one of the leading causes of mortality worldwide, according to the World Health Organization [1]. It exhibits diverse clinicopathological features, and its onset and progression are influenced by multiple risk factors. Prior pulmonary tuberculosis and concurrent active tuberculosis have been identified as independent risk factors, regardless of smoking status or chronic obstructive pulmonary disease [2,3]. Chai et al. demonstrated that *MKI67*, a gene common to tuberculosis and lung cancer, may serve as a key mediator of *Mycobacterium tuberculosis*-induced tumor cell proliferation, migration, and invasion [4]. Additionally, previous bioinformatic study has suggested that *EGFR*, *CECR2*, *LAMA3*, and *HSPA2* are potential genes associated with tuberculosis-related lung cancer [5]. However, the underlying molecular mechanisms of tuberculosis-associated lung cancer are not fully understood and warrant further investigation.

Exosomes are nanosized extracellular vesicles that mediate intercellular communication by transporting proteins, lipids, miRNAs, and mRNAs to recipient cells [6]. Their lipid bilayer protects this molecular cargo from degradation, enabling the stable transfer of biological signals that regulate inflammation, immune responses, and tumor progression within the pulmonary microenvironment [7,8]. Beyond their roles in intercellular communication and disease pathogenesis, exosomes and their molecular cargo have emerged as promising biomarkers for the diagnosis and monitoring of lung diseases. In particular, exosomal miRNAs are recognized as key regulators of cancer development by modulating the tumor microenvironment and have therefore attracted considerable attention as potential diagnostic biomarkers and therapeutic targets for cancer [9,10]. Exosomal miRNA influences not only the angiogenesis of lung cancer, induces epithelial-mesenchymal transition, and facilitates the reprogramming of the tumor microenvironment, but also impacts immunological regulation and the transmission of drug resistance, while participating in the regulation of lung cancer cell proliferation [11]. Exosomes are significantly concentrated in pleural effusions (PE) and serve as a valuable reservoir of disease-related molecular components, rendering them appealing biomarkers for lung cancer and tuberculosis [12]. Previous studies have reported that exosomal microRNAs derived from tuberculous pleural effusion not only mediate fibrosis but also promote the proliferation of lung cancer cells [13,14]. Tuberculous pleural effusion (TPE) derived miRNAs played important roles in pleural fibrotic remodeling by targeting key components of the TGF-β signaling pathway [14]. TPE-derived exosomes have been shown to enhance the malignant potential of lung cancer cells by promoting cell cycle progression through miR-130 and miR-423 [13].

We hypothesized that TPE-derived exosomal miRNAs may be associated with cancer-related genes and cellular processes relevant to cancer progression. To investigate this, we constructed a candidate miRNA–mRNA regulatory network by integrating exosomal miRNA profiling with transcriptomic analyses of xenograft tissues from an in vivo TPE-exosome-treated lung cancer xenograft model, thereby enabling the identification of functionally relevant downstream mRNA alterations. We then identified overlapping common genes (OCGs) by intersecting predicted exosomal miRNA target genes with candidate genes showing differential expression patterns in xenograft tissues. Finally, the relationships between selected candidate miRNAs and their corresponding target genes were experimentally assessed by Quantitative real-time reverse transcription PCR (qRT-PCR).

## 2. Materials and Methods

### 2.1. The Isolation and Quantification of Exosomes from Pleural Effusion

Pleural effusion (PE) samples were collected from patients at Chuncheon Sacred Heart Hospital during routine diagnostic thoracentesis. All human experiments were conducted in accordance with the Declaration of Helsinki. The collection and use of these samples were approved by the hospital’s Research Ethics Committee (Institutional Review Board number: 2012-27, approved on 5 June 2012). This study included two tuberculous pleural effusions (TPE) and two transudative pleural effusions (T). The demographic and clinical characteristics of the patients are summarized in Supplementary Table S1. Tuberculous effusion was defined as pleural fluid culture-positive for *Mycobacterium tuberculosis* that met the criteria for exudative PE. Transudative PE, defined according to Light’s criteria, was used as the control group [15]. Exosome isolation and characterization were performed and reported with reference to the relevant recommendations of the Minimal Information for Studies of Extracellular Vesicles 2023 (MISEV2023) guidelines [16]. Exosomes were isolated from PE samples using an Exo2d-EV isolation kit (Exosome Plus, Inc., Seoul, Republic of Korea) according to the manufacturer’s instructions. Samples were briefly centrifuged to remove cellular debris, followed by reagent-based exosome precipitation and resuspension in phosphate-buffered saline (PBS) for subsequent analyses. Exosomes were characterized by nanoparticle tracking analysis (NTA), transmission electron microscopy (TEM), and Western blot analysis using the positive exosomal markers CD63, CD9, and CD81, together with the negative marker Calnexin. Particle size distribution and concentration (particles/mL) were determined using the NanoSight NS300 system (NanoSight Ltd., Amesbury, UK). Morphological characterization was performed using field-emission transmission electron microscopy (FE-TEM; JEM-2100F, JEOL, Tokyo, Japan). Total RNA, extracted from the isolated exosomes using TRIzol reagent (Thermo Fisher Scientific, Waltham, MA, USA) as per the standard protocol, served as the basis for RNA analysis and sequencing.

### 2.2. Xenograft Model

Male BALB/c nude mice (7 weeks old, 18 ± 2 g; *n* = 10) were purchased from DooYeol Biotech (Seoul, Republic of Korea). All animals were housed under pathogen-free conditions (21–23 °C, 51–54% humidity, 12 h light/dark cycle) with free access to food and water. Then, the mice were maintained according to the United Kingdom Coordinating Committee on Cancer Research guidelines. For xenograft generation, 4 × 10^6^ A549 cells were subcutaneously transplanted into the flanks of 10 mice. Following tumor establishment, mice were randomly assigned to either the PBS group (*n* = 3) or the tuberculous pleural effusion-derived exosome group (*n* = 7) using a random number-based allocation method. Given the exploratory nature of the study, a larger number of animals was allocated to the TPE-exosome group to better capture potential variability in transcriptomic responses to TPE-derived exosome treatment. Tumor volumes, calculated as (tumor length × width^2^)/2. were monitored every other day through caliper measurement. Seven days after transplantation, when the tumors had reached a volume of 40 mm^3^, exosomes derived from tuberculous effusion were injected intratumorally at a dose of 100 μg per mouse every two days, for a total of seven injections. Animals were monitored daily, and predefined humane endpoints were applied in accordance with the Institutional Animal Care and Use Committee guidelines. On day 21, mice were euthanized under deep isoflurane anesthesia (3–5% for induction and 1.5–2.5% for maintenance in oxygen), followed by cervical dislocation to ensure death. Tumor measurements and subsequent molecular analyses were performed by investigators blinded to the treatment allocation. All procedures were approved by the Institutional Animal Care and Use Committee of Hallym University (no: Hallym 2020-21, approved on 12 August 2022). All animal experiments were conducted and reported in accordance with the ARRIVE guidelines.

### 2.3. In Vitro Experiment

The human lung adenocarcinoma cell line A549 (ATCC, Manassas, VA, USA) was cultured in RPMI 1640 medium (BYLABS, Hanam, Republic of Korea) supplemented with 10% fetal bovine serum, 100 U/mL penicillin, and 100 μg/mL streptomycin at 37 °C in a humidified incubator with 5% CO_2_. To investigate the correlation between miRNAs from TPE-derived exosomes and gene expression in lung cancer cells, A549 cells were transfected with miRNA mimics or inhibitors (200 pM) for hsa-miR-139, hsa-miR-224, hsa-miR-23b, hsa-miR-374a, hsa-miR-185, hsa-miR-191, and hsa-miR-155 (Bioneer, Daejeon, Republic of Korea). Transfections were performed using AccuFectTM transfection reagent (Bioneer) according to the manufacturer’s protocol, and transfection efficiency was assessed by qRT-PCR. To determine whether TPE-derived exosomes modulated the identified candidate miRNA–mRNA network, A549 cells were treated with TPE-derived exosomes, and the expression levels of the seven candidate miRNAs and five target genes (*RRAS2*, *RAC1*, *RAP1B*, *NOTCH2*, and *SMARCA4*) were subsequently examined by qRT-PCR. For co-treatment experiments, A549 cells were first transfected with the indicated miRNA mimics or inhibitors. Twenty-four hours after transfection, the cells were treated with TPE-derived exosomes. After an additional 18 h of incubation, cells were harvested for RNA extraction and qRT-PCR analysis of the corresponding target genes. To further evaluate the functional effects of candidate miRNA modulation in the presence of TPE-derived exosomes, wound-healing and Transwell invasion assays were performed under the same miRNA transfection and TPE-derived exosome treatment conditions. For the wound-healing assay, representative images were acquired at days 2 and 4, and wound closure was quantitatively analyzed at day 4. For the invasion assay, invaded cells were evaluated 48 h after treatment. Cells that had invaded through the membrane were stained with crystal violet, and the retained dye was quantified by measuring absorbance at 560 nm. For miRNA expression analysis, total RNA enriched for small RNAs was extracted from A549 cells treated with TPE-derived exosomes using the miRNeasy^®^ Mini Kit (QIAGEN, Hilden, Germany) according to the manufacturer’s protocol. Following RNA quantification, cDNA was synthesized using the Mir-X™ miRNA First-Strand Synthesis Kit (Clontech Laboratories, Inc., Mountain View, CA, USA). miRNA expression levels were subsequently analyzed by qRT-PCR and normalized to U6. Total RNA was extracted from A549 cells using the easy-BLUE™ Total RNA Extraction Kit (iNtRON Biotechnology, Seongnam, Republic of Korea), and cDNA was synthesized using the Maxime RT PreMix Kit (iNtRON Biotechnology, Seongnam, Republic of Korea). qRT-PCR was performed using SYBR Green I (Sigma-Aldrich, St. Louis, MO, USA) on a qTOWER3 G system (Analytik Jena GmbH, Jena, Germany). Relative gene expression levels were calculated using the 2^−ΔΔCt^ method, normalized to β-actin, and expressed relative to the control group. Primer sequences for the seven candidate miRNAs are listed in Supplementary Table S2, and those for the candidate target genes are listed in Supplementary Table S3.

### 2.4. Raw Data Processing and Differential Expression Analysis

miRNA and mRNA sequencing data were processed using separate bioinformatic pipelines to generate expression profiles. For miRNA analysis, FastQC (v0.11.9), MultiQC (v1.35) [17] and Cutadapt (v2.10) [18] were used to filter out low-quality reads and artificial sequences from the raw sequencing data. Reads containing at least the first five bases of the 3′ adapter sequence were identified as adapter-containing reads and trimmed accordingly. Only reads with a length of ≥18 bp after adapter trimming were retained for downstream analysis. In addition, the 3′ ends of reads were quality-trimmed using a Phred quality score cutoff of 10 (−q 10), and reads containing one or more ambiguous bases (‘N’) after trimming were excluded from further analysis. miRNA identification and quantification were performed using Bowtie 2 (v2.3.5.1) [19] and miRDeep2 (v2.0.0.8) [20]. Sequencing reads were aligned to reference miRNA sequences in miRBase (v22.1) [21], and DESeq2 (v1.36.0) [22] was used to normalize read counts and perform exploratory differential expression analysis. Candidate miRNAs showing differential expression patterns were selected using predefined thresholds of an adjusted *p* value (Benjamini-Hochberg correction) < 0.05 and |log2FoldChange| ≥ 1. For mRNA analysis, FastQC (v0.11.9), MultiQC (v1.35) and Trimmomatic (v0.39) [23] were used to filter low-quality reads and artificial sequences from the raw sequencing data in order to collect mRNA expression profiles. Trimmomatic was performed with the following parameters: adapter sequences were removed using TruSeq3-PE adapter clipping, bases with Phred quality scores < 3 were trimmed from the leading and trailing ends of reads, reads were trimmed when the average quality score within a 4-base sliding window fell below 15, and reads shorter than 36 bp after trimming were excluded from downstream analyses. To remove mouse-derived reads from the xenograft samples, cleaned reads were aligned separately to the human (GRCh38.p13) and mouse (GRCm39) reference genomes using the STAR (v2.7.10a) [24]. The resulting BAM files were analyzed with Disambiguate (v2018.05.03) [25], and only reads uniquely assigned to the human genome were retained for downstream analyses. Human-specific BAM files were sorted using SAMtools (v1.21) [26], and gene-level read counts were generated using HTSeq (v0.11.2) [27]. Differential expression analysis was performed using DESeq2 (v1.36.0), and candidate genes showing differential expression patterns were selected using a predefined threshold of an adjusted *p* value < 0.01. Principal Component Analysis (PCA) was performed using all detected genes for mRNA-seq and all detected miRNAs for miRNA-seq to assess sample-level variation. The three PBS-treated mRNA-seq samples showed relatively broad dispersion, with one sample positioned away from the others along PC1 (Supplementary Figure S1). However, all samples met the predefined Q30 thresholds (≥90% for mRNA-seq and ≥70% for miRNA-seq) and were therefore retained for downstream analyses.

### 2.5. Prediction of Candidate miRNA Target Genes and Identification of Overlapping Common Genes

We identified the target gene of candidate miRNA using two database of miRDB (v6.0, accessed on 25 July 2025, https://mirdb.org/) [28] and miRTarBase (v10.0, accessed on 25 July 2025, https://mirtarbase.cuhk.edu.cn/) [29]. We used only target genes with a prediction score higher than 80 from miRDB. We selected target genes present in both databases and overlapped them with the candidate genes derived from the xenograft transcriptomic analysis. The overlapped genes were referred to as overlapping common genes (OCGs).

### 2.6. Interaction Network Analysis

Among the OCGs, 5 genes were used for network analysis using Cytoscape (v3.10.4) [30] software. The five candidate genes were selected from OCGs using the following criteria: Candidate genes predicted to be targeted by two or more candidate miRNAs (*n* = 28). Genes showing inverse expression patterns relative to the corresponding miRNAs (increased miRNA–decreased gene expression or decreased miRNA-increased gene expression) (*n* = 23). Genes annotated in the COSMIC database (v104, accessed on 18 June 2026, https://cancer.sanger.ac.uk/cosmic/) [31] as oncogenes (upregulated), tumor suppressor genes (downregulated), or genes with dual roles (*n* = 5). This process prioritized *RRAS2*, *RAC1*, *RAP1B*, *NOTCH2*, and *SMARCA4* as candidate genes. Among the 27 candidate miRNAs, those predicted to regulate these five genes were incorporated into the Cytoscape network, yielding a candidate 7-miRNA–5-mRNA regulatory network. ClueGO (v2.5.10) [32] were used for functional analysis of the candidate genes showing differential expression patterns. GO terms include the 3-8 GO tree interval and a minimum of 2 genes that are associated with a term. These genes must represent at least 1% of the total number of associated genes. The STRING database (v11.0, accessed on 4 August 2026, https://version-11-0.string-db.org/) [33] was utilized to analyze the interactions between the candidate genes showing differential expression patterns.

### 2.7. Statistical Analysis

All statistical analyses were performed using GraphPad Prism software v8.02 (GraphPad Software Inc., San Diego, CA, USA) and home built scripts in R software v 4.2.0 [34]. Data are presented as the mean ± standard error of the mean (SEM). For comparisons between two independent groups, statistical significance was assessed using an unpaired two-tailed Student’s *t*-test. For comparisons involving three or more groups, one-way analysis of variance (ANOVA) followed by Bonferroni’s multiple comparison test for prespecified pairwise comparisons was used. Parametric analyses were performed under the assumption of approximate normality and homogeneity of variance. No data transformations were applied unless otherwise specified. For RNA-sequencing analyses, *p* values were adjusted for multiple testing using the Benjamini–Hochberg false discovery rate (FDR) method, as described in the RNA-sequencing analysis section. Group differences in the PCA were assessed using permutational multivariate analysis of variance (PERMANOVA) in the vegan package in R (v4.2.0). The corresponding R^2^ and *p* values are reported in Supplementary Figure S1. For other experiments involving multiple group comparisons, multiplicity was controlled using the indicated Bonferroni post hoc test. All tests were two-sided, and *p* < 0.05 was considered statistically significant.

## 3. Results

### 3.1. Characterization of Tuberculous Pleural Effusion-Derived Exosomes and Their Protumorigenic Activity In Vivo

**Figure 1:**
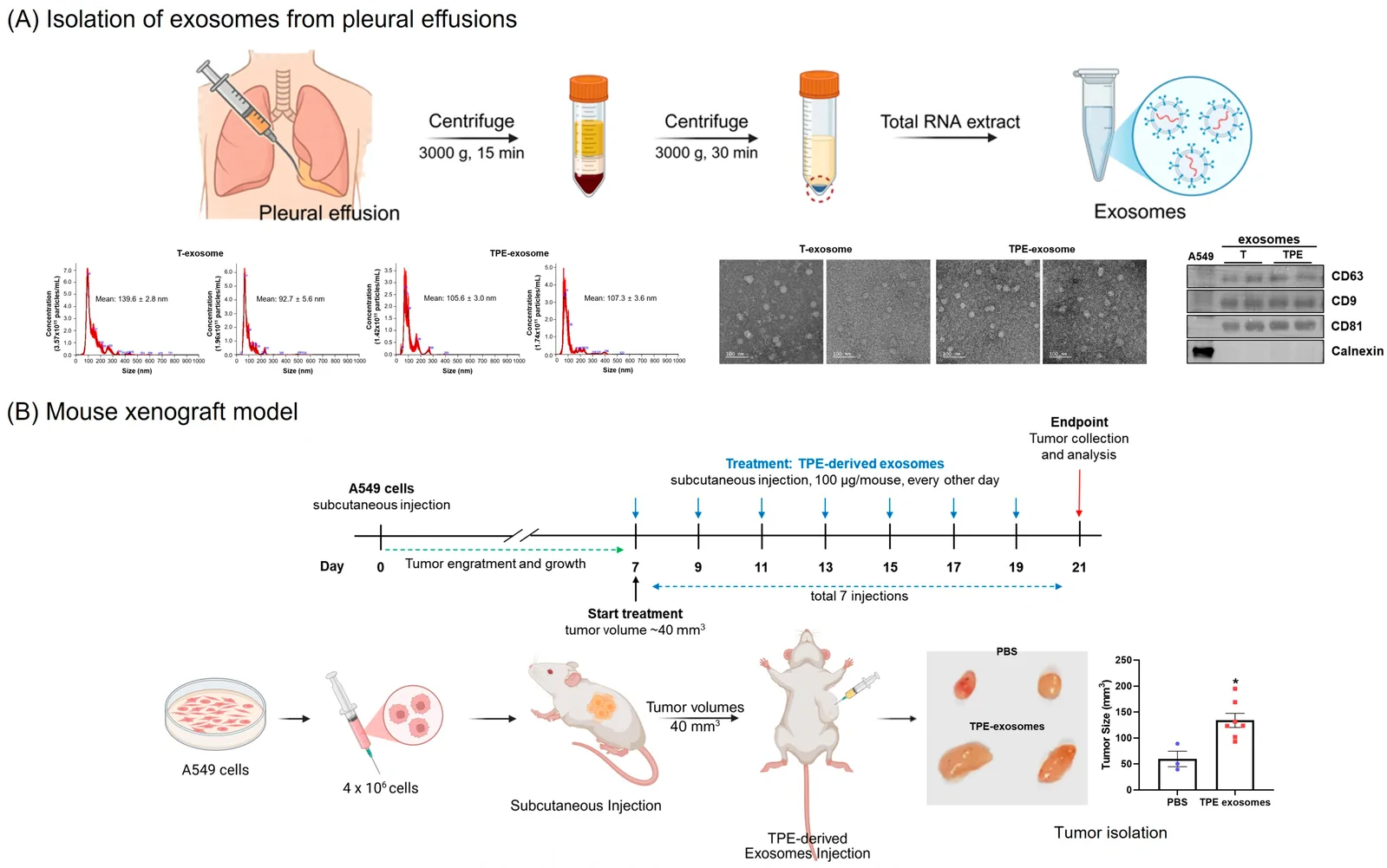
Schematic overview of the experimental design. (**A**) Isolation of tuberculous pleural effusion (TPE)-derived exosomes. Exosomes were isolated from two TPE samples (*n* = 2) and compared with two transudative pleural effusion (T) samples (*n* = 2). The isolated exosomes were characterized by nanoparticle tracking analysis (NTA), transmission electron microscopy (TEM), and Western blot analysis using exosomal markers. (**B**) Mouse xenograft model treated with TPE-derived exosomes. A549 xenograft-bearing mice were randomly assigned to the PBS group (*n* = 3) or the TPE-derived exosome-treated group (*n* = 7). Intratumoral administration of TPE-derived exosomes was initiated 7 days after tumor implantation and continued every 2 days for a total of seven injections. Tumor growth was monitored during the subsequent 14-day treatment period. On day 21, mice were euthanized, tumors were excised, tumor size was measured using digital calipers, and tumor tissues were collected for RNA sequencing. Final tumor volumes are presented as mean ± SEM. Statistical significance between the PBS and TPE-derived exosome-treated groups was determined using an unpaired two-tailed Student’s *t*-test. * *p* < 0.05 vs. PBS group.

Figure 1 summarizes the overall experimental scheme. To identify miRNAs differentially expressed in exosomes derived from tuberculous pleural effusion (TPE), exosomes were isolated from two tuberculous effusions and two transudative pleural effusions (Figure 1A). Exosome identity was confirmed by NTA, TEM, and Western blot analysis of established exosomal markers. NTA analysis showed that exosomes isolated from the two TPE samples and two transudative pleural effusion sample exhibited a typical size distribution, with mean particle diameters of 105.6 ± 3.0 nm, 107.3 ± 3.6 nm, 139.6 ± 2.8 nm and 92.7 ± 5.6 nm, respectively. TEM confirmed the characteristic cup-shaped morphology of the isolated vesicles, and Western blot analysis confirmed the expression of the exosomal markers CD9, CD63, and CD81, whereas the negative marker Calnexin was not detected (Figure 1A).

To evaluate the functional effects of TPE-derived exosomes on lung cancer progression, an in vivo mouse xenograft tumor model was employed (Figure 1B). Xenograft tumors receiving intratumoral injections of TPE-derived exosomes were significantly larger than those treated with PBS (126.49 ± 11.71 mm^3^ vs. 59.72 ± 15.01 mm^3^, *p* = 0.014).

### 3.2. Exploratory Analysis of miRNA and Gene Expression Patterns

**Figure 2:**
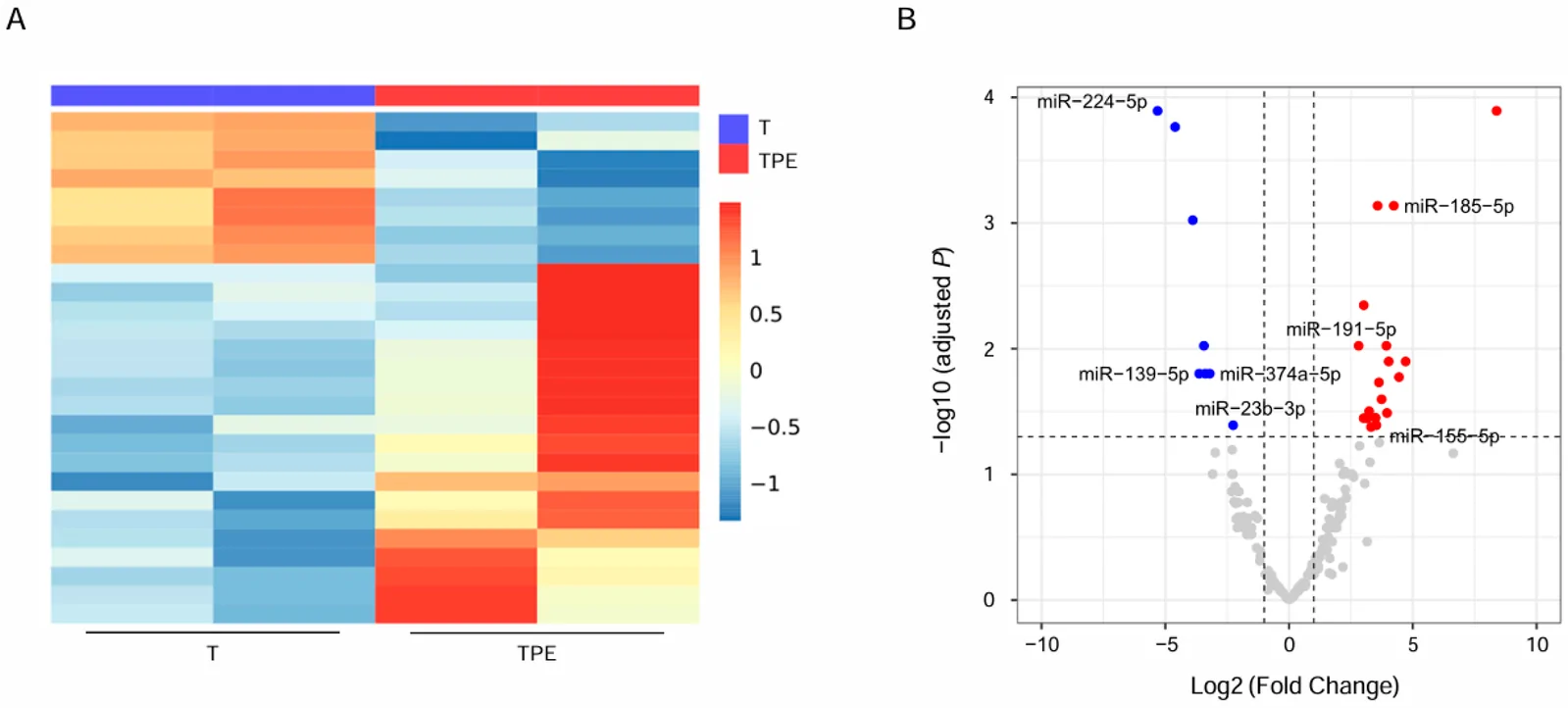
Exploratory analysis of miRNA expression patterns in TPE-derived exosomes. (**A**) Heatmap and (**B**) volcano plot of miRNA sequencing data illustrating expression patterns between TPE-derived and transudative pleural effusion-derived exosomes. Candidate miRNAs showing differential expression patterns were selected using predefined cutoff criteria of |log_2_ fold change| ≥ 1 and adjusted *p* < 0.05. TPE (*n* = 2) and transudative pleural effusion (*n* = 2).

**Figure 3:**
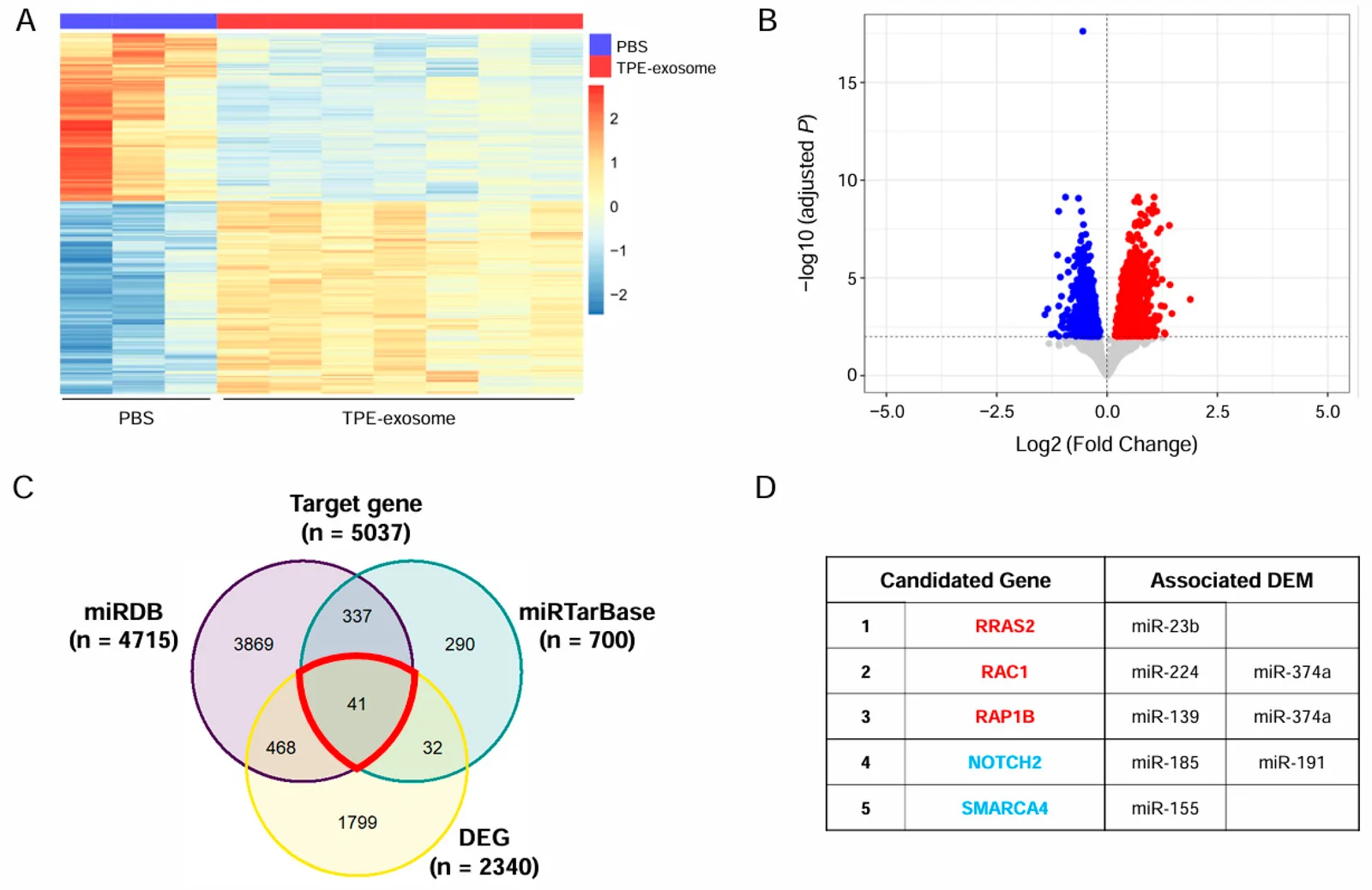
Integrated analysis of candidate gene expression patterns and overlapping common genes (OCGs) in xenograft tumors treated with TPE-derived exosomes. (**A**) Heatmap and (**B**) volcano plot of transcriptomic sequencing data illustrating gene expression patterns in TPE-exosome-treated xenograft tumors compared with PBS-treated controls. Candidate genes showing differential expression patterns were selected using the predefined cutoff criterion of adjusted *p* < 0.01. (**C**) Venn diagram illustrating the overlap between candidate genes from the xenograft transcriptomic analysis and predicted target genes of candidate miRNAs, as determined using the miRDB and miRTarBase databases. (**D**) Candidate genes prioritized from the OCGs and the corresponding miRNAs predicted to regulate these genes, illustrating putative miRNA–mRNA regulatory relationships. Xenograft mRNA-seq: TPE-exosome group (*n* = 7) and PBS group (*n* = 3).

The miRNA expression profiles of exosomes derived from tuberculous pleural effusion (TPE) and transudative pleural effusion were analyzed. Differential expression analysis using DESeq2 with an adjusted *p*-value (Benjamini–Hochberg correction) < 0.05 and an absolute log2 fold change (|log2FoldChange|) ≥ 1 identified 27 candidate miRNAs showing differential expression patterns between the transudate and TPE groups. Of these, 19 showed increased expression (miR-1290, miR-4449, miR-1285-3p, miR-185-5p, miR-106b-3p, miR-378a-3p, let-7i-3p, miR-143-3p, miR-339-3p, miR-142-5p, miR-155-5p, miR-23a-5p, miR-8485, miR-320a-3p, miR-140-3p, miR-130b-3p, miR-423-5p, miR-320b, and miR-191-5p), whereas 8 showed decreased expression (miR-23b-3p, miR-374a-5p, miR-4271, miR-10a-5p, miR-139-5p, miR-125b-5p, miR-30a-3p, and miR-224-5p) (Figure 2; Supplementary Table S4). Similarly, exploratory RNA-seq analysis of xenograft tumors identified 2340 candidate genes showing differential expression patterns after species disambiguation to retain human-specific reads (Figure 3A,B; Supplementary Table S5). Given the limited sample sizes, particularly in the miRNA-seq analysis, these expression patterns should be considered exploratory and hypothesis-generating and require validation in larger independent datasets.

### 3.3. Prioritization of Candidate Genes from OCGs

Target genes of the candidate miRNAs were predicted using miRDB and miRTarBase, yielding 4715 and 700 predicted target genes, respectively. As shown in Figure 3C, these predicted target genes were compared with candidate genes showing differential expression patterns in the xenograft transcriptomic analysis, resulting in 41 overlapping common genes (OCGs). Five candidate genes were subsequently prioritized from the 41 OCGs using a stepwise filtering strategy based on the following criteria: (1) genes predicted to be targeted by two or more candidate miRNAs (*n* = 28); (2) genes showing inverse expression patterns relative to the corresponding miRNAs (increased miRNA–decreased gene expression or decreased miRNA-increased gene expression) (*n* = 23); and (3) genes annotated in the COSMIC database as oncogenes showing increased expression, tumor suppressor genes showing decreased expression, or genes with dual roles (*n* = 5). This stepwise analysis prioritized *RRAS2*, *RAC1*, *RAP1B*, *NOTCH2*, and *SMARCA4* as five candidate target genes. Based on target prediction and the observed expression patterns, candidate miRNA–mRNA regulatory relationships were proposed, including miR-23b–RRAS2, miR-224–RAC1, miR-374a–RAC1, miR-139–RAP1B, miR-374a–RAP1B, miR-185–NOTCH2, miR-191–NOTCH2, and miR-155–SMARCA4 (Figure 3D, Supplementary Table S6).

### 3.4. Experimental Assessment of Candidate miRNA–mRNA Regulatory Relationships

**Figure 4:**
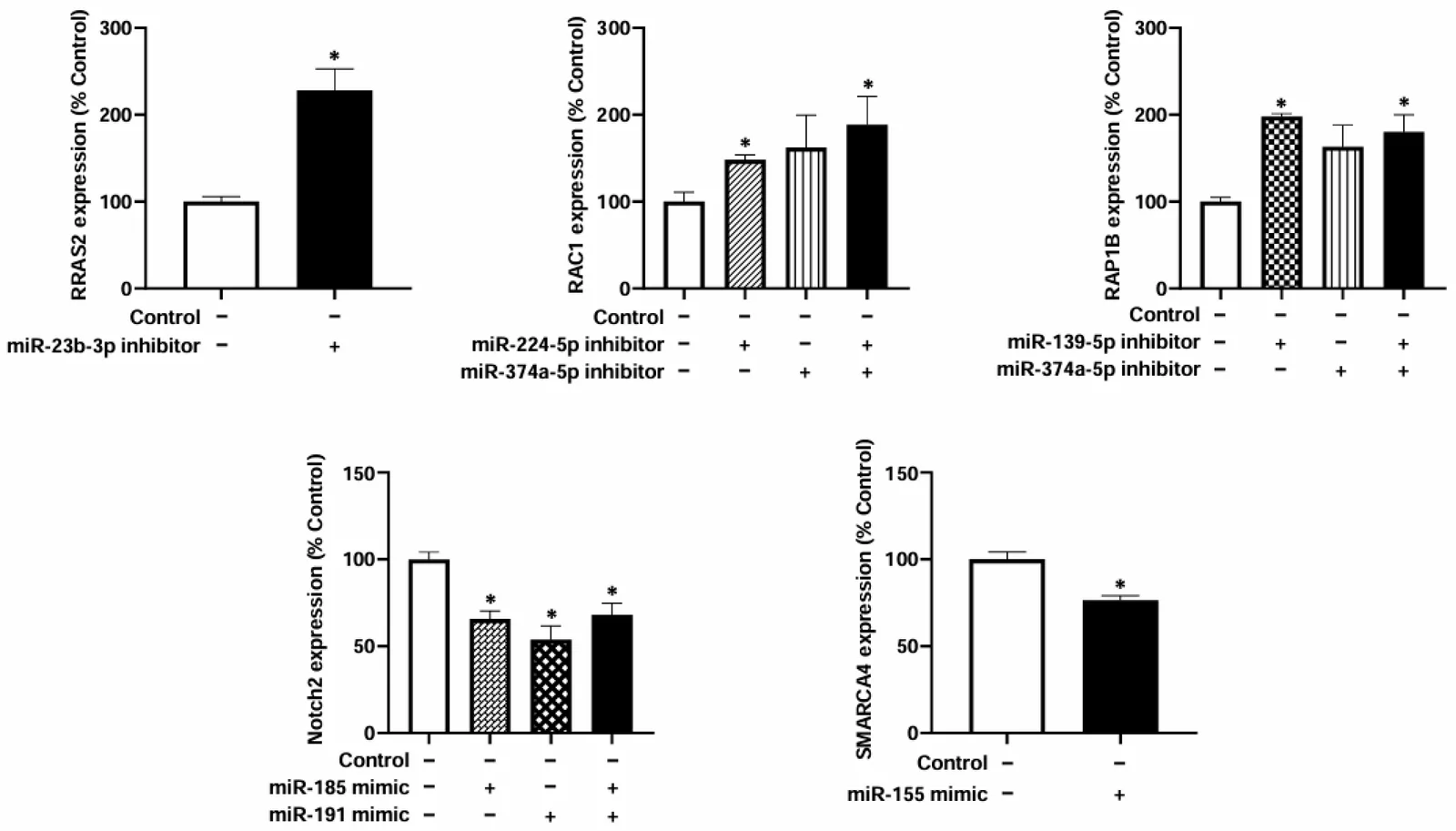
In vitro assessment of target gene expression following transfection with candidate miRNA mimics or inhibitors. A549 cells were transfected with miRNA mimics or inhibitors corresponding to the predicted miRNA–mRNA pairs (miR-23b–RRAS2, miR-224/miR-374a–RAC1, miR-139/miR-374a–RAP1B, miR-185/miR-191–NOTCH2, and miR-155–SMARCA4). Eighteen hours after transfection, cells were harvested, and the mRNA expression levels of the predicted target genes were quantified by qRT-PCR. Relative expression levels were normalized to β-actin. Data are presented as mean ± SEM from three independent experiments (*n* = 3). Statistical significance was determined using an unpaired two-tailed Student’s *t*-test. * *p* < 0.05 vs. Control.

To experimentally assess the candidate miRNA–mRNA regulatory relationships prioritized through the integrated analyses, A549 cells were transfected with four miRNA inhibitors and three miRNA mimics (Figure 4). A significant upregulation of *RRAS2* expression was observed in cells transfected with the miR-23b-3p inhibitor. Inhibition of miR-224-5p and miR-374a-5p led to a significant upregulation of *RAC1* expression. Notably, simultaneous inhibition of both miRNAs resulted in a greater increase in target gene expression compared with single-miRNA inhibition. In addition, RAP1B expression increased following inhibition of miR-139-5p and miR-374a-5p. Conversely, overexpression of miR-185 and miR-191 led to a significant downregulation of NOTCH2 compared with the control group. Furthermore, overexpression of miR-155 markedly suppressed *SMARCA4* expression. To determine whether TPE-derived exosomes modulate the identified candidate network in lung cancer cells, A549 cells were treated with TPE-derived exosomes, and the expression levels of the seven candidate miRNAs and five corresponding target genes were examined by qRT-PCR. TPE-derived exosome treatment altered the expression of the candidate miRNAs and target genes (Supplementary Figure S2). Consistent with the RNA-seq findings, qRT-PCR analysis showed similar directional changes in the five target genes following TPE-derived exosome treatment, with increased expression of *RRAS2*, *RAC1*, and *RAP1B* and decreased expression of *NOTCH2* and *SMARCA4*. However, the magnitude of these changes and their statistical significance varied among individual genes. To further assess whether the candidate miRNA–mRNA relationships were maintained in the presence of TPE-derived exosomes, A549 cells were transfected with the indicated miRNA mimics or inhibitors and subsequently treated with TPE-derived exosomes. The expression patterns of the corresponding target genes were then evaluated by qRT-PCR. Modulation of the candidate miRNAs altered target-gene expression relative to the TPE-derived exosome-treated group, providing additional experimental support for the proposed regulatory relationships (Supplementary Figure S3). The functional relevance of these regulatory effects was further examined using wound-healing and Transwell invasion assays under TPE-derived exosome treatment. Modulation of the candidate miRNAs altered the migratory and invasive capacities of A549 cells compared to the TPE-derived exosome-treated group, consistent with the observed target-gene expression changes (Supplementary Figures S4 and S5). Collectively, these findings are consistent with the proposed candidate regulatory relationships involving miR-23b-3p-RRAS2, miR-224-5p/miR-374a-5p-RAC1, miR-139-5p/miR-374a-5p-RAP1B, miR-185/miR-191-NOTCH2, and miR-155/SMARCA4 following TPE-derived exosome exposure.

### 3.5. Integrated Network and Functional Enrichment Analysis of Candidate Genes

**Figure 5:**
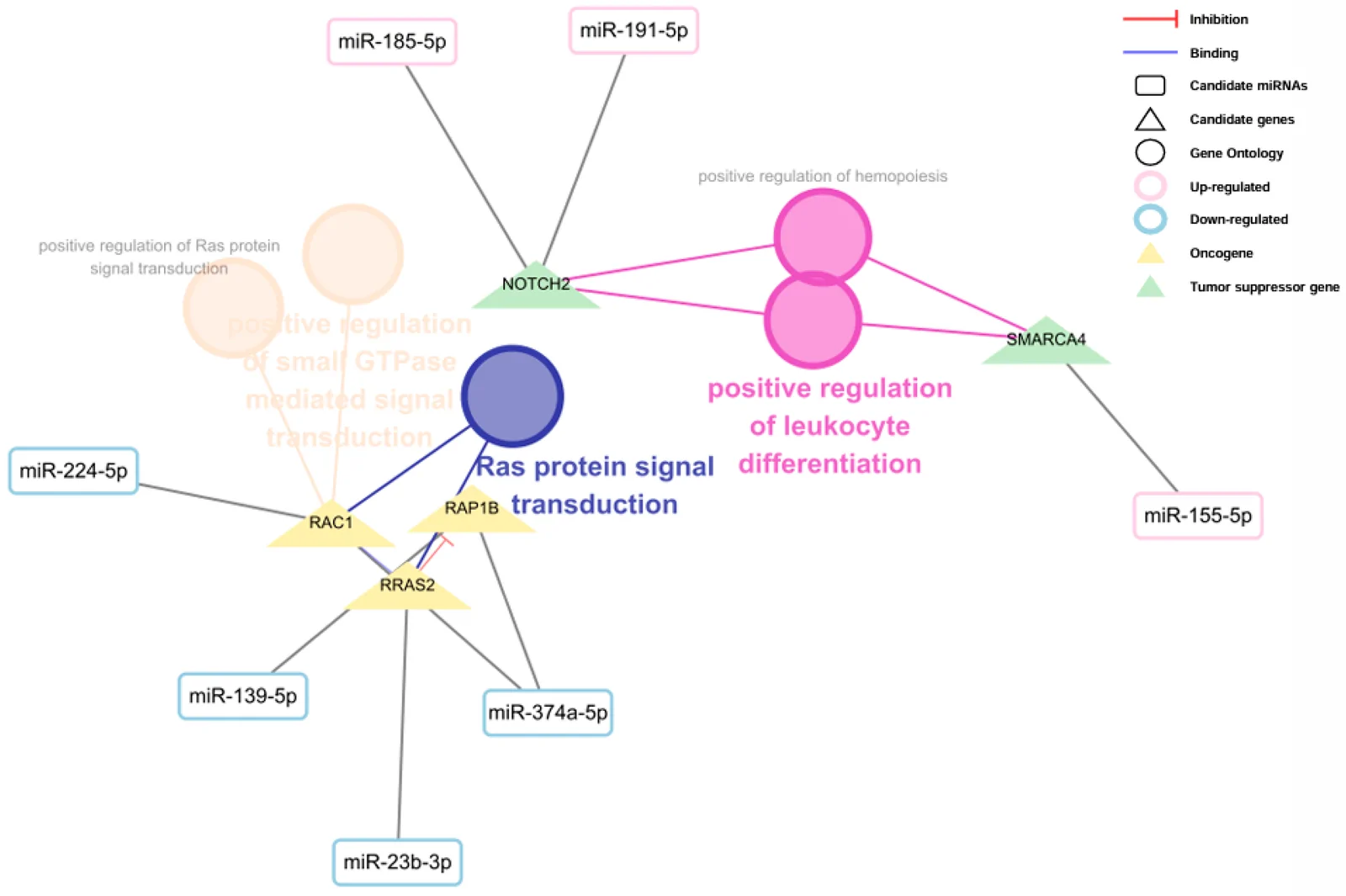
Candidate miRNA–mRNA regulatory network and functional relationships among the prioritized genes. The network was constructed and visualized using Cytoscape software. Candidate miRNAs showing increased and decreased expression patterns are indicated in pink and blue, respectively. Oncogenes are shown in yellow, and tumor suppressor genes are shown in green. miRNA-mRNA inhibitory interactions are shown as red edges, and binding interactions are shown as blue edges. Functional associations among the prioritized genes were obtained from the STRING database.

To further characterize the potential biological relationships among the candidate miRNAs and genes, integrated network and functional enrichment analyses were performed (Figure 5). Functional enrichment analysis suggested that *RAC1*, *RAP1B*, and *RRAS2* were associated with Ras protein signal transduction, whereas *NOTCH2* and *SMARCA4* were associated with the positive regulation of leukocyte differentiation. Network analysis further indicated potential associations among these candidate genes. Collectively, these analyses provide additional biological context for the candidate miRNA–mRNA regulatory network.

## 4. Discussion

Modulation of the immunological microenvironment after *Mycobacterium tuberculosis* infection is essential for both effective treatment of tuberculosis (TB) and prevention of disease recurrence, according to recent studies [35,36]. Specifically, a deeper understanding of the immune response within tuberculous granulomas is increasingly recognized as important for the development of novel host-directed therapeutic approaches. Accumulating epidemiological and experimental evidence also supports a link between tuberculosis and lung cancer [37,38,39,40]. Long-term immunological and inflammatory changes in the pulmonary microenvironment following tuberculosis may influence the development and course of lung cancer. These changes in the microenvironment may be maintained and propagated through intercellular communication, in which exosomes serve as important mediators of signaling among resident, immune, and tumor cells. Exosomes can additionally alter the pulmonary milieu by affecting immune cell activity, macrophage polarization, fibroblast activation, angiogenesis, and extracellular matrix remodeling, thus aiding in the formation of a protumorigenic niche [41,42]. Characterizing exosome-mediated miRNA–mRNA regulatory networks may therefore provide a basis for investigating potential molecular relationships between TB-associated alterations in the pulmonary microenvironment and lung cancer. Accordingly, we aimed to explore potential miRNA–mRNA regulatory relationships using a TPE-exosome-treated lung cancer xenograft model.

Our exploratory analysis suggested that miR-374a, miR-224, miR-139, and miR-23b showed decreased expression patterns in TPE-derived exosomes, whereas miR-185, miR-191, and miR-155 showed increased expression patterns. These findings are generally consistent with previous reports regarding tuberculosis-associated miRNA expression patterns [43]. For example, miR-374a-5p has been reported to be downregulated in the blood of patients with tuberculosis [43,44] and miR-224 likewise exhibited decreased expression in the plasma of patients with active tuberculosis [45,46]. Zhang et al. have reported that hsa-miR-23b-3p, a predicted target of genes associated with multidrug-resistant tuberculosis, is significantly downregulated in MDR-TB patients compared with drug-sensitive TB patients and healthy controls [47]. Upregulated miR-155, miR-185, and miR-191 have been implicated as important regulators of host immune responses during *Mycobacterium* tuberculosis infection, particularly in processes such as inflammation, apoptosis, autophagy, and immune cell activation [48,49,50].

In addition to their varied expression, these miRNAs possess established biological roles in cancer and immunological modulation [51]. In non-small cell lung cancer (NSCLC), miR-155 is a well-established oncomiR involved in inflammatory signaling, immune-cell activation, and tumor progression, whereas miR-191 has been reported to exhibit predominantly oncogenic functions. In contrast, miR-374a-5p, miR-23b-3p and miR-139-5p are widely recognized as tumor-suppressive miRNAs [52]. The biological functions of miR-224-5p and miR-185, however, appear to be context dependent and may vary across different cancer types.

Previous studies have suggested overlapping pathogenic, immunological, and molecular landscapes between tuberculosis and lung cancer [53,54]. Several shared microRNAs (miR-155, miR-146a, miR-29a, miR-30a, miR-125b, and let-7) have been identified in both diseases; these may regulate common immune-related signaling pathways associated with autophagy and apoptosis [53]. Although the biological activities of these miRNAs have been separately investigated in tuberculosis and lung cancer, their potential relationships within exosome-mediated miRNA–mRNA networks remain incompletely understood. Our findings provide a candidate framework for exploring potential relationships between TPE-derived exosomal miRNAs and genes involved in cancer- and inflammation-related pathways.

In this study, we explored a candidate miRNA–mRNA regulatory network associated with TPE-derived exosome exposure by integrating predicted target genes of exosomal miRNAs with candidate genes derived from the xenograft model. Candidate miRNA–mRNA relationships were prioritized using complementary target information from miRDB and miRTarBase, which provide computational predictions and experimentally supported miRNA–target information, respectively. This integrative approach enabled the prioritization of candidate miRNA–mRNA relationships for further investigation. Among these candidates, five genes were selected based on the predefined criteria, and their potential regulatory relationships with the corresponding miRNAs were further examined through in vitro experimental analyses.

Among the candidate relationships identified in our analysis, those involving miR-155–SMARCA4 and miR-185/miR-191–NOTCH2 may be of particular biological interest. *SMARCA4*, the catalytic subunit of the SWI/SNF chromatin-remodeling complex, has previously been shown to be regulated by miR-155 through binding to its 3′ untranslated region (3′UTR) [55,56]. *SMARCA4* deficiency occurs in approximately 8% of NSCLCs and has been associated with chemoresistance, early recurrence, and poor clinical outcomes [57]. NOTCH signaling is a highly conserved pathway that plays critical roles in cell fate determination, proliferation, differentiation, and apoptosis [58], although its role in lung cancer is context-dependent [59]. *NOTCH2* has been suggested to exert differentiation-associated and potentially tumor-suppressive functions in lung adenocarcinoma [59]. Consistent with our network analysis, *NOTCH2* and *SMARCA4* were associated with the positive regulation of leukocyte differentiation. These observations provide a basis for further investigation of their potential roles in cancer- and inflammation-related processes following TPE-derived exosome exposure.

Our analysis also identified candidate relationships involving miR-23b-3p–RRAS2, miR-224-5p/miR-374a-5p–RAC1, and miR-139-5p/miR-374a-5p–RAP1B. *RRAS2*, *RAC1*, and *RAP1B* are representative components of Ras-related protein signal transduction pathways involved in cell proliferation, cytoskeletal remodeling, adhesion, migration, invasion, and immune regulation [60,61]. Aberrant activation of *RRAS2* and *RAC1* has been associated with oncogenic signaling, therapeutic resistance, and poor clinical outcomes in NSCLC [62,63], whereas *RAP1B* contributes to VEGF-A-mediated immunosuppressive signaling and maintenance of cancer stem cell-like properties [64,65].

Previous studies have linked the five selected genes to biological pathways related to cancer and immune regulation. *RRAS2* and *RAC1* have been linked to oncogenic signaling pathways, including PI3K/Akt and MAPK, which play important roles in lung cancer growth, survival, and metastatic behavior [61,62]. In addition, *NOTCH2*, a key component of Notch signaling, and *SMARCA4* have been associated with epithelial plasticity and epithelial-mesenchymal transition (EMT)-related processes [66,67]. Although these previously reported associations provide biological context for the potential involvement of the prioritized genes in cancer- and inflammation-related processes, they should not be interpreted as evidence of pathway activation in our study or as establishing a direct mechanistic link to tuberculosis-associated lung carcinogenesis. Taken together, our findings suggest potential associations between miRNAs carried by TPE-derived exosomes and candidate genes with previously reported roles in cancer- and inflammation-related signaling pathways. Given the stability of exosomal miRNAs in biological fluids, these candidate relationships may warrant further investigation to determine their biological relevance.

This study has several important limitations despite identifying candidate miRNA–mRNA regulatory relationships associated with TPE-derived exosome exposure through in silico analyses and in vitro validation. First, the exosomal miRNA discovery analysis included only two tuberculous pleural effusion (TPE) samples and two transudative pleural effusion samples. Given the small number of donors, demographic and clinical heterogeneity between the TPE and transudative groups may have influenced the observed exosomal miRNA profiles. Therefore, validation in larger and better-matched independent clinical cohorts is required. In addition, the use of a precipitation-based method for exosome isolation may have resulted in the co-isolation of non-vesicular extracellular components, potentially affecting the identified miRNA profiles. The in vitro experiments were also conducted using a single lung cancer cell line (A549), which limits the generalizability of the findings across different lung cancer models.

Second, the xenograft RNA-seq analysis was based on a limited sample size, particularly in the PBS control group, which reduced the statistical power for detecting gene expression differences. Accordingly, the transcriptomic findings should be considered exploratory and hypothesis-generating. Furthermore, we used a subcutaneous BALB/c nude mouse xenograft model rather than clinical tumor specimens or an orthotopic lung cancer model. Because this model does not include *Mycobacterium tuberculosis* infection, it does not recapitulate the native pulmonary microenvironment or the chronic inflammatory and immunological milieu of tuberculosis-associated lung carcinogenesis. Although the TPE-exosome co-treatment experiments provided additional support for the candidate regulatory relationships following TPE-derived exosome exposure, TPE-exosome exposure cannot reproduce this complex milieu. In addition, the lack of functional T cells in BALB/c nude mice precluded evaluation of adaptive immune responses. Although species-disambiguation analysis was performed to minimize the contribution of mouse-derived reads, residual mouse-derived contamination in the xenograft transcriptomic data cannot be completely excluded.

Third, the use of COSMIC annotation as a prioritization criterion inherently favored genes already recognized as cancer-related. Therefore, the selected genes should not be interpreted as newly identified cancer-associated genes, but rather as known cancer-related genes prioritized within a candidate miRNA–mRNA regulatory network in the context of TPE-exosome exposure. Finally, although several predicted miRNA–mRNA regulatory relationships were experimentally examined in vitro, the validation strategy primarily relied on changes in mRNA expression following miRNA mimic or inhibitor transfection. While these findings support the proposed regulatory relationships, they do not establish direct miRNA–target interactions and cannot exclude indirect effects mediated through other downstream targets or regulatory pathways.

Further studies are therefore warranted to establish the biological and clinical significance of these candidate regulatory networks, including direct mechanistic validation of miRNA–mRNA interactions, validation in larger and better-matched clinical cohorts incorporating malignant pleural effusions without tuberculosis as an additional disease control, evaluation in multiple lung cancer cell lines, and the use of more physiologically relevant models, including immunocompetent and active tuberculosis models.

Future studies integrating single-cell and spatial transcriptomics with multi-omics analyses and circulating exosome profiling may provide a more comprehensive understanding of the potential molecular links between tuberculosis and lung cancer. Furthermore, artificial intelligence-assisted biomarker discovery may facilitate the identification of candidate biomarkers and molecular targets for future investigation, thereby supporting the development of precision diagnostic and therapeutic approaches.

In conclusion, our integrative in silico analyses and preliminary experimental assessment prioritized a candidate miRNA–mRNA regulatory network associated with TPE-derived exosome exposure in lung cancer models. The prioritized genes included *NOTCH2* and *SMARCA4*, which are related to leukocyte differentiation, and *RAC1*, *RAP1B*, and *RRAS2*, which are associated with Ras signaling. These findings provide a basis for further investigation of the biological relevance of these relationships in more physiologically pertinent models that better reflect the tuberculosis-associated microenvironment.

## Data Availability

The mRNA-sequencing data of xenografted tumor generated in this study have been deposited in the European Nucleotide Archive (ENA) at EMBL-EBI under accession number PRJEB124263.

## Supplementary Materials

The following supporting information can be downloaded at https://www.mdpi.com/article/10.3390/biomedicines14092060/s1, Table S1: Clinical characteristics of the study participant; Table S2: Human sequences and assession numbers for miRNA primers used for the experiments; Table S3: List of PCR primer used for the experiments; Table S4: DESeq2 results of differentially expressed miRNAs (|log2FoldChange| ≥ 1, adjusted *p* < 0.05) between Transudate and TPE; Table S5: DESeq2 results of differentially expressed genes (adjusted *p* values < 0.01) between Control and TB; Table S6: Selection of potential candidate genes from 41 overlapping common genes (OCGs) based on three filtering criteria (*n* = 5). Figure S1: Principal component analysis (PCA) of the samples. Figure S2: TPE-derived exosomes modulate the expression of candidate miRNAs and their predicted target genes in A549 cells. Figure S3: Validation of candidate miRNA–mRNA interactions under TPE-derived exosome stimulation. Figure S4: Effects of candidate miRNA modulation on the migration and invasion of A549 cells in the presence of TPE-derived exosomes. Figure S5: Uncropped Western blot images for exosomal marker characterization.

## Abbreviations

The following abbreviations are used in this manuscript:

TPE	Tuberculous pleural effusion
OCGs	Overlapping common genes
PE	Pleural effusion
T	Transudative pleural effusions
PBS	Phosphate-buffered saline
NTA	Nanoparticle tracking analysis
TEM	Transmission electron microscopy
